# Morphological features of 52 cases of breast phyllodes tumours with local recurrence

**DOI:** 10.1007/s00428-022-03383-8

**Published:** 2022-07-29

**Authors:** Jiaxin Bi, Hongping Tang, Xiao Lin, Xuewen Yu, Yingying Liang, Lu Zhang, Zhixin Li, Mumin Shao

**Affiliations:** 1 Department of Pathology, Shenzhen Traditional Chinese Medicine Hospital, The Fourth Clinical Medical College of Guangzhou University of Chinese Medicine, No.1 Fuhua Road, Futian District, Shenzhen, MD518033 Guangdong China; 2 Affiliated Shenzhen Maternity & Child Healthcare Hospital, Southern Medical University, Shenzhen, Guangdong Province, China; 3 Department of Surgery, Shenzhen Traditional Chinese Medicine Hospital, The Fourth Clinical Medical College of Guangzhou University of Chinese Medicine, Shenzhen, Guangdong Province, China

**Keywords:** Breast phyllodes tumour, Pathological diagnosis, Morphological feature

## Abstract

Typical phyllodes tumours (PTs) of the breast are fibroepithelial neoplasms characterised histologically by stromal hypercellularity and leaf-like structures. However, morphological changes may be atypical in some cases, increasing the difficulty of diagnosis and the likelihood of misdiagnosis. To provide more morphological clues for pathological diagnosis of PTs, we retrospectively analysed 52 PT cases with typical morphological features after recurrence, and summarized the clinicopathological characteristics of the paired primary tumours. We found five special histological features in the primary tumours distinct from classic PTs, namely (1) PTs with epithelioid feature (three cases); (2) PTs with gland-rich feature (eight cases); (3) PTs with fibroadenoma-like feature (20 cases); (4) PTs with myxoid fibroadenoma-like feature (five cases); and (5) PTs with pseudohemangiomatoid stromal hyperplasia–like feature (four cases). All the features can exist independently, and a few cases displayed more than two distinctive features at the same time. In this cohort of recurrent PTs, all the primary tumours were absent of recognisable stromal hypercellularity and leaf-like structures that are the critical diagnostic criteria of PTs; however, they showed some other non-classic characteristics which may provide significant clues for the diagnosis of PTs. Particularly, tumours with epithelioid feature displayed high grade at earlier stages, tumours with fibroadenoma-like feature were most likely to be confused with classical fibroadenomas, and tumours with myxoid feature were prone to be neglected because of their hypocellularity.

**Supplementary Information:**

The online version contains supplementary material available at 10.1007/s00428-022-03383-8.

## Introduction

A phyllodes tumour (PT) is a fibroepithelial neoplasm of the breast. In Asian countries, the incidence of phyllodes tumours (PTs) is high and has a tendency to occur in young women. PTs usually locally recur within 2–3 years of diagnosis, with an overall incidence of 21%; the rate is 10–17% for benign, 14–25% for borderline, and 23–30% for malignant tumours [1]. Furthermore, while overall survival has been shown to be reduced to 30 months in patients with metastases [2], many studies have shown that the complete resection of a tumour with a negative margin is an effective way to prevent tumour recurrence [3, 4]. Several histological features have been used for the diagnosis and grading of PTs, including the tumour border, stromal cellularity, stromal atypia, mitotic activity, stromal overgrowth, and malignant heterologous elements[5]. However, there are no definitive criteria or clear cutoffs for each histological parameter. Tumour heterogeneity is an additional factor leading to diagnostic dilemmas. In one study, 10 breast pathologists were invited to make a diagnosis of 21 cases of challenging cellular fibroepithelial lesions. In nine cases (43%), the diagnosis varied and included fibroadenoma (FA) and borderline PTs, with the tumour grading of a single PT case, ranging from benign to malignant [5]. Rakha et al. also found low consistency between pathologists in determining the grade of PTs, especially for borderline PTs (42%) [6]. Moreover, various biomarkers were selected to assist in tumour diagnosis, such as CD34, vascular endothelial growth factor, β-catenin, B cell leukaemia/lymphoma 2 (Bcl-2), epidermal growth factor receptor (EGFR), and p53 [7–9]. Positivity and specificity were, however, not optimal. For example, only 37 to 57% of the malignant PTs were positive for CD34 [7].

Currently, the presence of hypercellularity and stromal fronds are considered to be essential PT diagnostic indices according to the WHO Classification of Breast Tumours [10]; however, whether all lesions or early-stage lesions have these features is not clear and has not been thoroughly studied yet. In line with this, we conducted a retrospective study of PTs with local recurrence from 52 female patients with typical PT morphological changes after recurrence, to examine the morphologically variant features worthy of attention since these may be potential early PT diagnostic clues.

## Materials and methods

### Patient selection

Fifty-two female patients diagnosed with PT after local recurrence but not diagnosed with PT at the first surgical resection were selected as study participants. By reviewing the clinical history and ultrasound examination, the location of each breast tumour in the same patient was verified to be consistent. The patient age, tumour size, surgery time, and original and recurrent pathological diagnoses were recorded. Forty-four of these patients were from our own institution (Shenzhen Traditional Chinese Medicine Hospital), and eight of them were referrals from the Affiliated Shenzhen Maternity & Child Healthcare Hospital, Southern Medical University. In addition, we collected 50 cases of typical fibroadenoma (FA) (form 2010 to 2012) without recurrence for morphological comparison with PTs resembling FAs.

### Pathological review

All the haematoxylin–eosin (H&E) slides of the surgical specimen were reviewed and examined for histological features by two experienced pathologists. Tumour border was categorised as well-defined or permeative. Stromal cellularity and stromal atypia were graded into mild, moderate, and marked. Stromal overgrowth was defined by the absence of epithelial elements in one low-power microscopic filed (40; ZEISS AXIO Scope.A1; field area 0.26mm^2^). Mitotic count was defined as the number of mitotic figures per square millimetre. The tumours were graded into benign, borderline, and malignant based on the WHO criteria [10]. Additional histologic features, including the presence of the sharp boundary between interlobular and intralobular stroma, leaf-like fronds, myxoid changes, hyalinization, vascular hyperplasia, pseudoangiomatous stromal hyperplasia (PASH) in the stroma, and ductal epithelial hyperplasia in the glands, were also assessed in the primary tumours and FAs.

### Immunohistochemistry

Immunohistochemical studies (CD34, Bcl-2, and EGFR) were performed on all the formalin-fixed paraffin–embedded sections using an automated stainer (Leica Bond-III, Australia), with a heat-based antigen retrieval technique with BOND Epitope Retrieval Solution 2 (prediluted, pH 9.0) for 20 min at 100 °C. The ready-to-use antibodies against CD34 (QBEnd/10), Bcl-2 (MX022), and EGFR (EP22) were purchased from MXB Biotechnologies (Labvision). For EGFR, immunoreactivity with a moderate to strong staining intensity in ≥ 10% of the neoplastic cells was considered positive; for CD34 and Bcl-2, > 10% of neoplastic cells with unequivocal staining were considered positive reactivity.

### Statistical analyses

All statistical analyses were carried out using SPSS V27.0 (IBM, Armonk, NY, USA). Fisher’s exact test was used for categorical variables, and the Kruskal–Wallis test was used for non-parametric variables.

## Results

### Patient information

The information of the 52 female patients diagnosed with PT with a history of recurrence is summarized in Table 1 with the details provided in Supplementary Table 1. At first diagnosis, the median patient age was 31.5 (range 16–51) years, the mean tumour size was 3.2 (range 1.0–9.7) cm, and the location of the tumour was on the right side in 30 cases. The first recurrence occurred on average of 36.4 (median 24.5, range 3–114) months after the first surgical resection. In 42 patients, the local recurrence occurred only once, while 10 patients experienced two or more times. In total, there were 17 benign PTs, 24 borderline PTs, and 11 malignant PTs in the recurrent tumours.

**Table 1:** Summary of clinicopathological features

Clinicopathological parameters	*N* = 52 (%)
Age (median) (y)	31.5 (range 16–51)
Size (mean) (cm)	3.20 (1.0–9.7)
Location	
Left	20 (38.46)
Right	30 (57.69)
Bilateral	2 (3.85)
Recurrence interval time (median) (m)	24.5 (range 3–114)
Recurrence	
Once	42 (80.77)
Twice	9 (17.31)
Thrice	1 (1.92)
Tumour grade of the last surgery	
Benign	17 (32.69)
Borderline	24 (46.15)
Malignant	11 (21.15)

### Variant morphological features in the primary tumours

As compared to the recurrent tumours, the primary tumours showed five variant features that were completely different from those in the classic PTs (namely epithelioid, gland-rich, FA-like, myxoid, and PASH). The histological characteristics are shown in Table 2 and Supplementary Table 2.

**Table 2:** Clinicopathological parameters of primary PTs with different features

	Epithelioid feature	Gland-rich feature	FA-like feature	Myxoid feature	PASH feature	Classic feature	*P* value
	*N* = 3 (%)	*N* = 8 (%)	*N* = 20 (%)	*N* = 5 (%)	*N* = 4 (%)	*N* = 12 (%)	
Age (median, years)	21 (18–47)	33 (22–40)	30.5 (19–46)	36 (35–50)	30.5 (26–39)	25 (16–51)	0.242^*^
Interval time (median, months)	10 (8–14)	26 (13–92)	20.5 (3–95)	26 (11–82)	67.5 (31–71)	34 (4–114)	0.092^*^
Location (*n*)							0.330
Left	2 (66.67%)	5	4	2	3	4	
Right	1 (33.33%)	3	14	3	1	8	
Bilateral	0	0	2	0	0	0	
Tumour size (mean, cm)	4.8 (2–9)	4.3 (2.3–9.7)	3.1 (1.5–5.5)	3.1 (1–6)	2.4 (1.3–3)	2.6 (1.2–4)	0.378^*^
Initial tumour grade							
Benign	0	5 (62.5%)	17 (85%)	2 (40%)	4 (100%)	10 (83.33%)	0.013
Borderline	2 (66.67%)	3 (37.5%)	3 (15%)	3 (60%)	0	2 (16.67%)	
Malignant	1 (33.33%)	0	0	0	0	0	
Tumour upgrade	2 (66.67%)	5 (62.5%)	9 (45%)	3 (60%)	2 (50%)	7 (58.33%)	0.955
Frequency of recurrence							0.145
Once	3 (100%)	7 (87.5%)	18 (90%)	4 (80%)	3 (75%)	7 (58.33%)	
Twice	0	1 (12.5%)	2 (10%)	0	1 (25%)	5 (41.67%)	
Thrice	0	0	0	1 (20%)	0	0	
Tumour border							< 0.01
Well-defined	0	7 (87.5%)	20 (100%)	1 (20%)	3 (75%)	10 (83.33%)	
Permeative	3 (100%)	1 (12.5%)	0	4 (80%)	1 (25%)	2 (16.67%)	
Stromal features							
Cellularity							0.504
Mild	3 (100%)	6 (75%)	13 (65%)	5 (100%)	4 (100%)	10 (83.33%)	
Moderate	0	2 (25%)	7 (35%)	0	0	2 (16.67%)	
Marked	0	0	0	0	0	0	
Atypia							< 0.01
None	0	5 (64.5)	13 (65%)	0	0	0	
Mild	0	1 (12.5%)	7 (35%)	5 (100%)	4 (100%)	11 (91.67%)	
Moderate	2 (66.67%)	2 (25%)	0	0	0	1 (8.33%)	
Marked	1 (16.67%)	0	0	0	0	0	
Overgrowth							NA
Absent	3 (100%)	8 (100%)	20 (100%)	5 (100%)	4 (100%)	12 (100%)	
Present	0	0	0	0	0	0	
Mitoses/mm^2^							0.026
Absent	0	2 (25%)	4 (20%)	0	3 (75%)	0	
Present (≥ 1)	3 (100%)	6 (75%)	16 (80%)	5 (100%)	1 (25%)	12 (100%)	
Enlargement of epithelial–stromal junction							< 0.01
Absent	3 (100%)	0	0	5 (100%)	3 (75%)	2 (16.67%)	
Present	0	8 (100%)	20 (100%)	0	1 (25%)	10 (83.33%)	
Leaf-like fronds							NA
Absent	3 (100%)	8 (100%)	20 (100%)	5 (100%)	4 (100%)	0	
Present	0	0	0	0	0	12 (100%)	
Myxoid changes							0.002
Absent	3 (100%)	8 (100%)	15 (75%)	0	4 (100%)	9 (75%)	
Present	0	0	5 (25%)	5 (100%)	0	3 (25%)	
Hyalinisation							0.002
Absent	3 (100%)	8 (100%)	14 (70%)	5 (100%)	0	11 (91.67%)	
Present	0	0	6 (30%)	0	4 (100%)	1 (8.33%)	
Vessel proliferation							< 0.01
Absent	3 (100%)	2 (25%)	5 (25%)	0	4 (100%)	0	
Present	0	6 (75%)	15 (75%)	5 (100%)	0	12 (100%)	
PASH							0.009
Absent	3 (100%)	7 (87.5%)	10 (50%)	5 (100%)	0	7 (58.33%)	
Present	0	1 (12.5%)	10 (50%)	0	4 (100%)	5 (41.67%)	
Epithelial hyperplasia							< 0.01
Absent	3 (100%)	0	7 (35%)	5 (100%)	0	0	
Present	0	8 (100%)	13 (65%)	0	4 (100%)	12 (100%)	

^*^Kruskal–Wallis test; all other values were calculated with Fisher’s exact test. *FA*, fibroadenoma; *PT*, phyllodes tumour; *PASH*, pseudohemangiomatoid stromal hyperplasia; *NA*, not applicable

#### PTs with epithelioid feature (three cases)

**Fig. 1:**
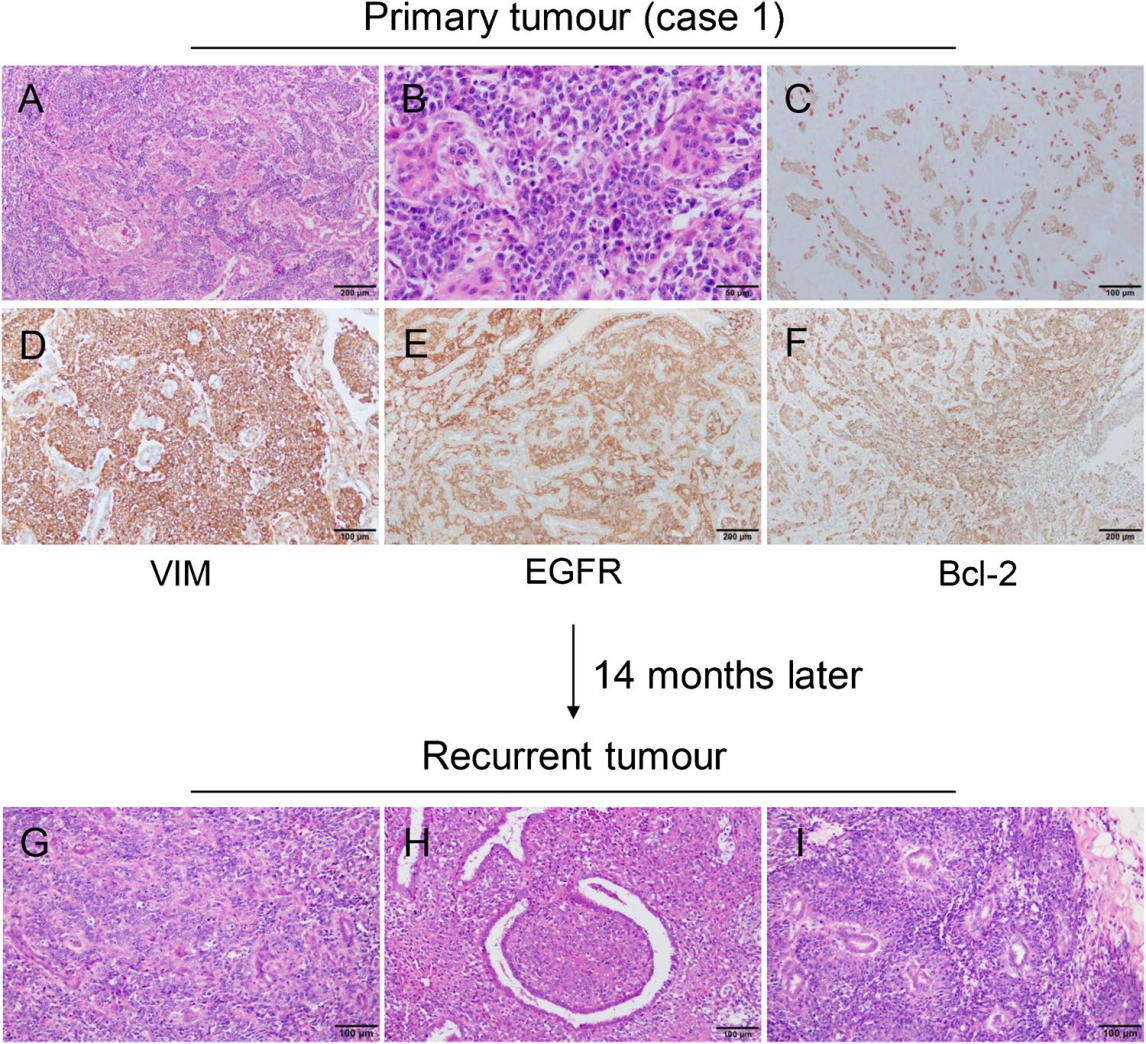
Phyllodes tumours with epithelioid feature. **a, b** Atypical tumor cells intermingled with non-atypical glandular epithelium, mimicking invasive growth pattern. **c** Double staining of CK8 (brown) and p63 (red) demonstrating intact myoepithelial surrounding the glands. **d, e, f** Neoplastic stromal cells are diffusely positive for VIM, EGFR, and Bcl-2. **g** Epithelial pseudo-infiltration is still visible. **h** Recognisable leaf-like frond is observed. **i** ‘Multi-layered rosette’ structure appeared in some area

A mixed distribution of stromal and glandular components mimicking infiltrating carcinoma was observed in three cases of PTs with epithelioid feature. The neoplastic stromal cells surrounded the irregular glands tightly and shared the morphological characteristics of epithelial cells (sparse cytoplasm, large round or quasi-round nuclei, and obvious nucleoli) (Fig. 1A, B). The outline of the glands was distorted with intact myoepithelial layer that was confirmed with double staining using p63 and CK8 (Fig. 1C). Tumour cells were stained strongly with VIM, EGFR, and Bcl-2 (Fig. 1D–F). No typical leaf-like stromal structure was found in the primary tumours; however, the presence of nuclear pleomorphism and the brisk mitotic activity of the stromal cells supported the diagnosis of a malignant PT. After recurrence, it was still difficult to separate the tumour cells from non-neoplastic glands (Fig. 1G), but the expansion of the stroma into a leaf-like structure was apparent (Fig. 1H). Moreover, the epithelioid tumour cells were arranged radially around the gland to form a ‘multi-layered rosette’ structure in the recurrent tumour of case one (Fig. 1I). The diagnosis was made as malignant PTs.

#### PTs with gland-rich feature (eight cases)

**Fig. 2:**
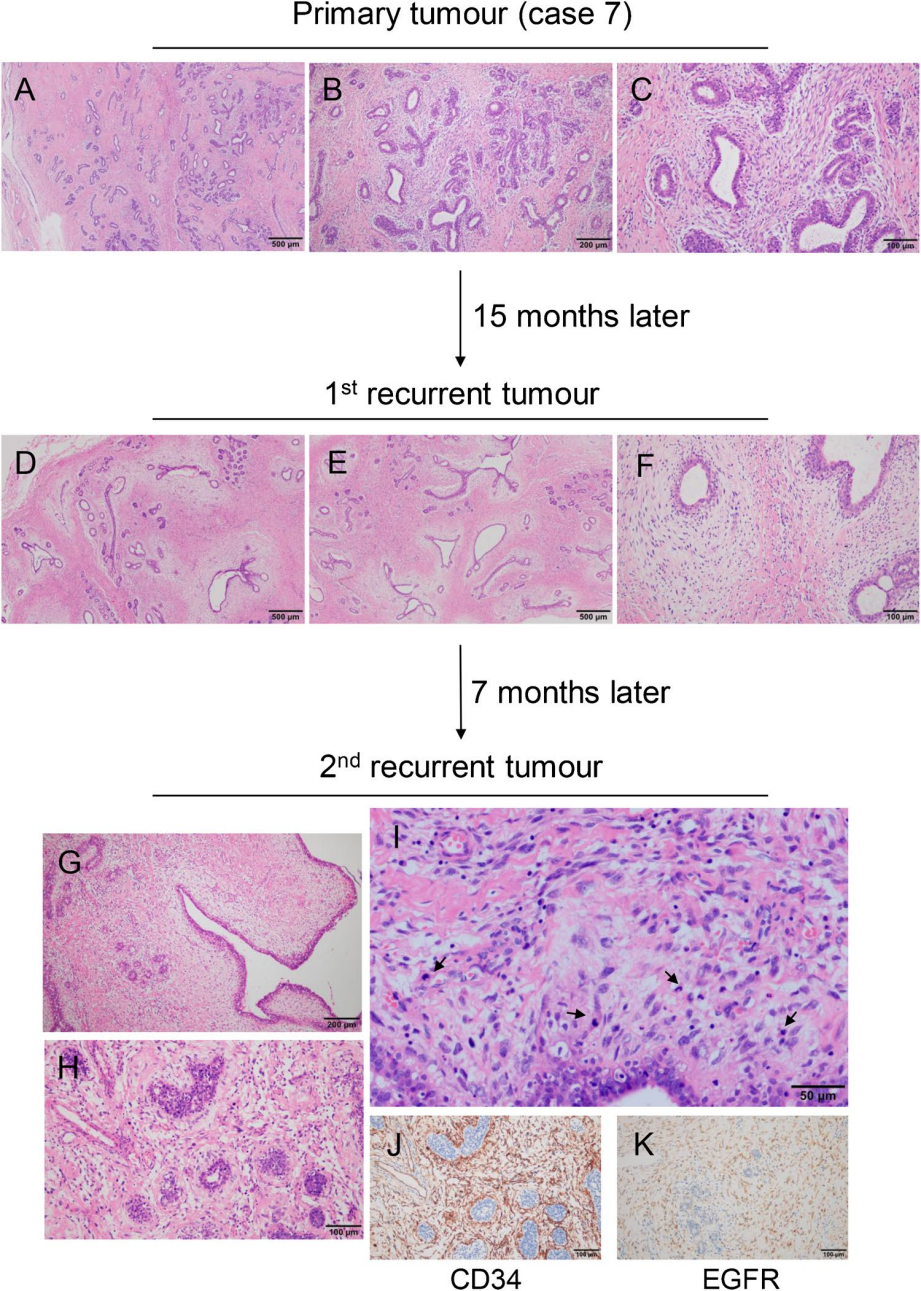
Phyllodes tumours with gland-rich feature. **a** Abundant glands and ambiguous lobules are observed in primary tumour. **b, c** Interlobular stromal increased slightly without cellular atypia. **d, e** A clear epithelial–stromal junction appeared around the ducts. **f** Stromal cells show mild to moderate atypia. **g** Leaf-like projections extended into dilated elongated lumina. **h** PASH-like feature is observed in some area. **i** Cell atypia with brisk mitotic activity (arrow). **j, k** CD34 and EGFR are diffusely positive

Five cases were diagnosed as breast adenosis at the first mastectomy because of the presence of abundant glands, visible terminal duct lobular unit (TDLU) structures, and absence of excessive stromal growth (Fig. 2A, B). Nevertheless, compared with the adjacent normal TDLUs, the epithelial–stromal junction around the glands was obvious, the staining became lighter, and the cell density increased slightly without any obvious atypia (Fig. 2C). After local recurrence, the structure of the TDLU was vaguely visible; however, the area of interlobular stroma expanded, and even formed a leaf-like structure with the spindle cells showing mild to moderate atypia (Fig. 2D–K). Two cases were diagnosed as cellular FA due to the mildly abundant stromal cells but lack of leaf fronds. For the recurrent tumours, three malignant, three borderline, and two benign PTs were confirmed.

#### PTs with FA-like feature (20 cases)

**Fig. 3:**
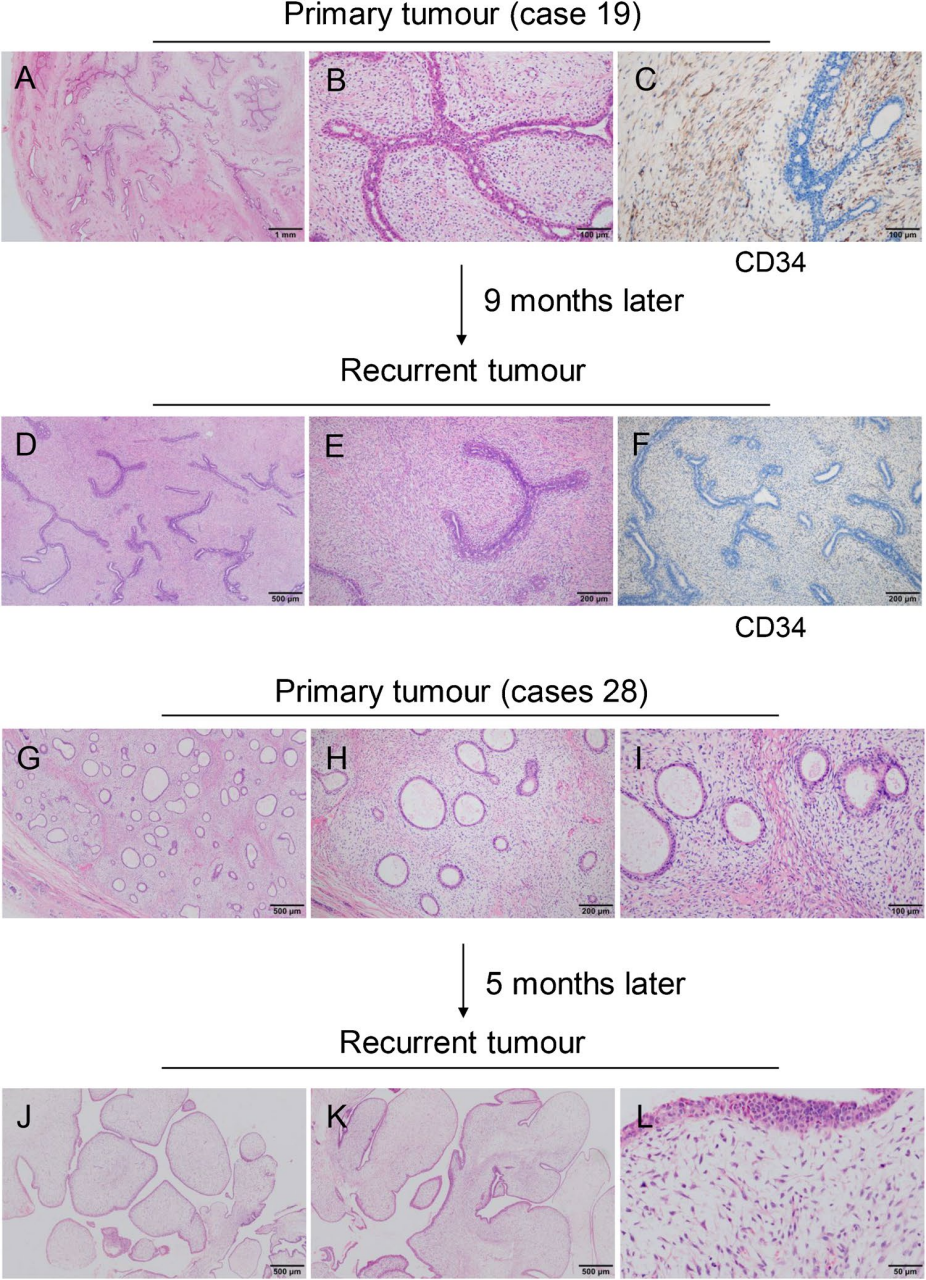
Phyllodes tumours with FA-like feature. **a** Predominantly intracanalicular growth pattern with low cell density. **b** Mild hyperplasia of stromal cells around individual slit-like ducts. **c** Stromal cells are positive for CD34. **d, e** Stromal cell overgrowth squeezing the duct into a cleft. **f** Tumour cell loss of CD34 expression. **g, h** Pericanalicular growth pattern. **i** Intralobular stroma shows mild hyperplasia. **j, k** Leaf-like structures. **l** Stromal cells show atypia and mitosis

All the 20 cases that were diagnosed as FA after the first mastectomy showed two growth pattern similar to FA: intracanalicular and pericanalicular patterns. For the intracanalicular pattern, the expansion of the stromal cells compressed the ducts into slit-like spaces. The intralobular stromal area was clearly demarcated from the interlobular stroma and its staining was lighter, but without any cell atypia evident. After recurrence, the tumour grade progressed to borderline or malignant in six cases, exhibiting the overgrowth and atypia of stromal cells and the formation of typical leaf-like structures (Fig. 3A–F). For the pericanalicular pattern, the stromal cells grew around the patent ducts accompanied by the expansion of the epithelial–stroma junction (Fig. 3G–L). Three cases displayed marked cellular atypia and increased mitotic counts after recurrence.

#### PTs with myxoid feature (five cases)

**Fig. 4:**
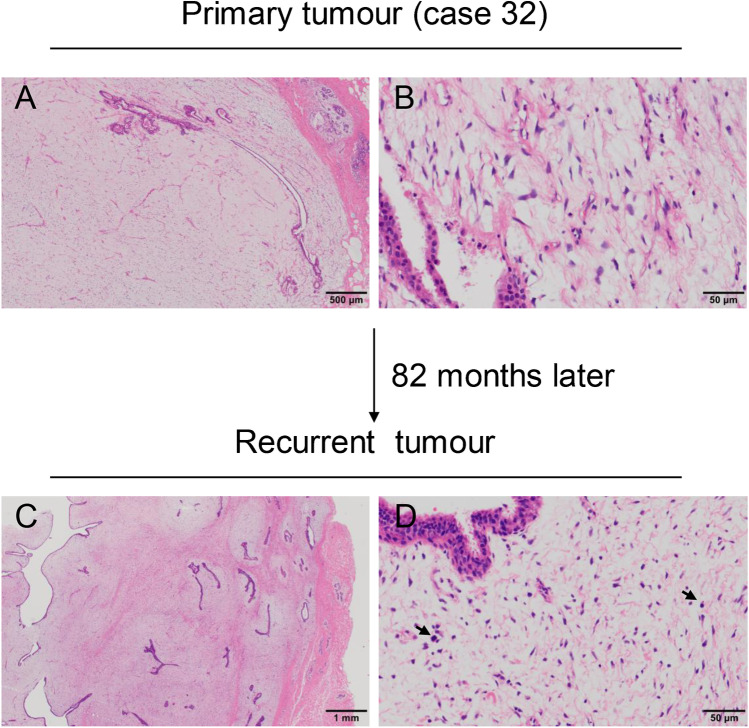
Phyllodes tumours with myxoid feature. **a** Prominent myxoid matrix with curvilinear blood vessels in the tumour and adjacent TDLU. **b** Hypocellular appearance with spindled or stellate tumour cells. **c** The ducts are distended and compressed by hypocellular myxoid stroma. **d** Mitotic figures can be observed at higher power (arrow)

A group of PTs had myxoid changes as the most significant feature, with irregular tumour borders instead of pushing edges. The tumour cells were sparse and wavy, with rope-like collagen and branched thin-walled blood vessels in the background. The interlobular interstitial region disappeared. The stromal area in the adjacent TDLU also showed myxoid degeneration (Fig. 4A, B). Definite mitosis was observed in three primary tumours in a high-power flow. After recurrence, the mucus was distributed diffusely, with mucus lakes forming, and the density of tumour cells was still low and had insignificant atypia; however, brisk mitosis was observed in all five cases (Fig. 4C, D).

#### PTs with PASH feature (four cases)

**Fig. 5:**
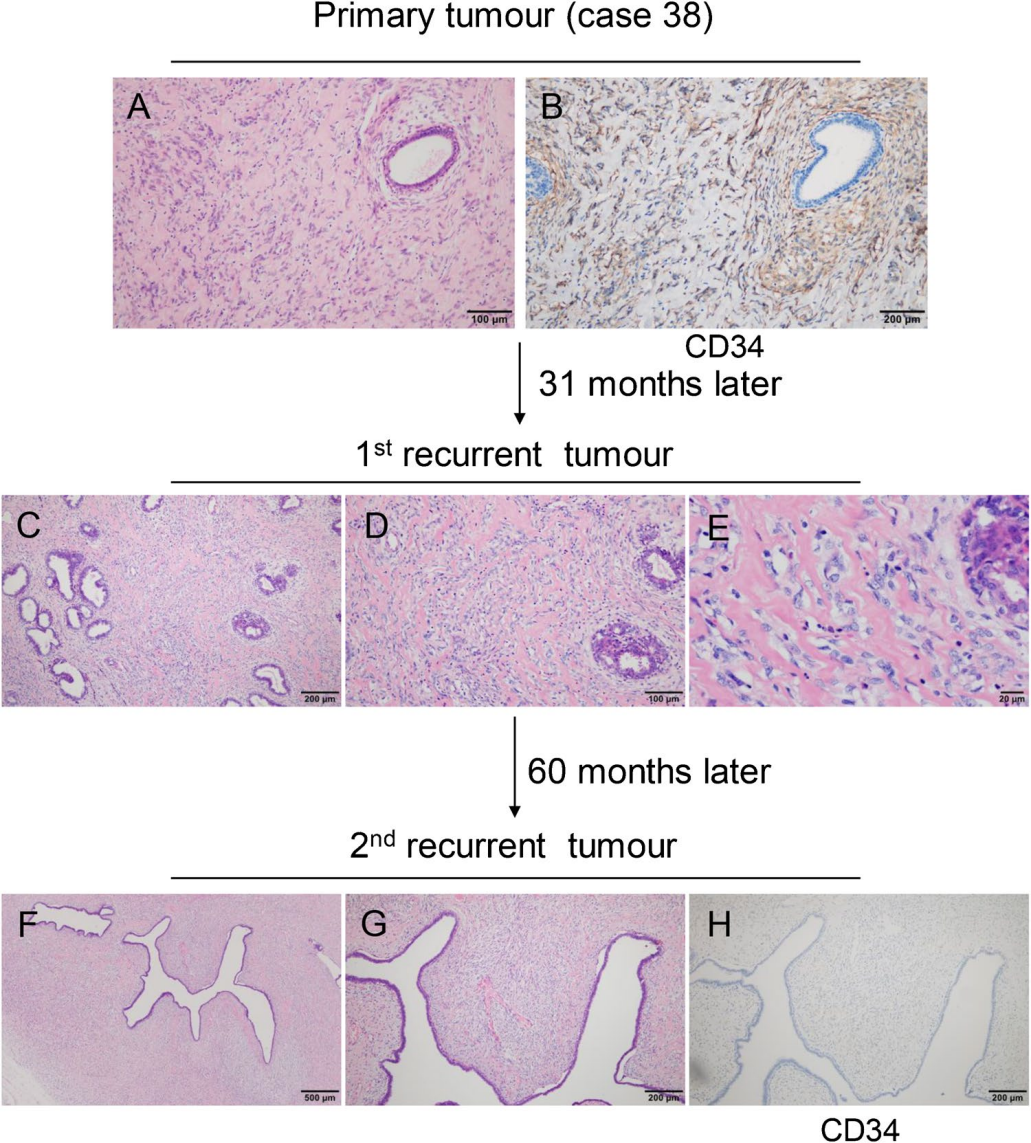
Phyllodes tumours with PASH feature. **a** Pseudoangiomatous stroma arranged in fascicular pattern. **b** Stromal cells are positive for CD34. **c, d** Hyperplasia in stroma and epithelium. **e** Stromal cells display atypia. **f, g** Typical leaf-like structures with hypercellularity in stroma. **h** CD34 is negative in tumour cells

The primary tumours were diagnosed as FA with PASH morphology in the stromal area or PASH after the first resection. Interlobular stromal cells proliferated with vascular-like spaces diffusely, but with no red blood cells. The spaces were lined with bland flattened cells with abundant collagen production, and there was a diffuse expression of CD34 (Fig. 5A,B). Collagen products were seen in the stromal area. Following the second resection, the tumours progressed with stromal cell proliferation, a decrease in the collagen products, and the formation of leaf-like structures (Fig. 5C-H). Finally, two cases were diagnosed with borderline grade tumours due to the presence of increased cellular atypia and mitotic count.

#### PTs with classic feature (12 cases)

**Fig. 6:**
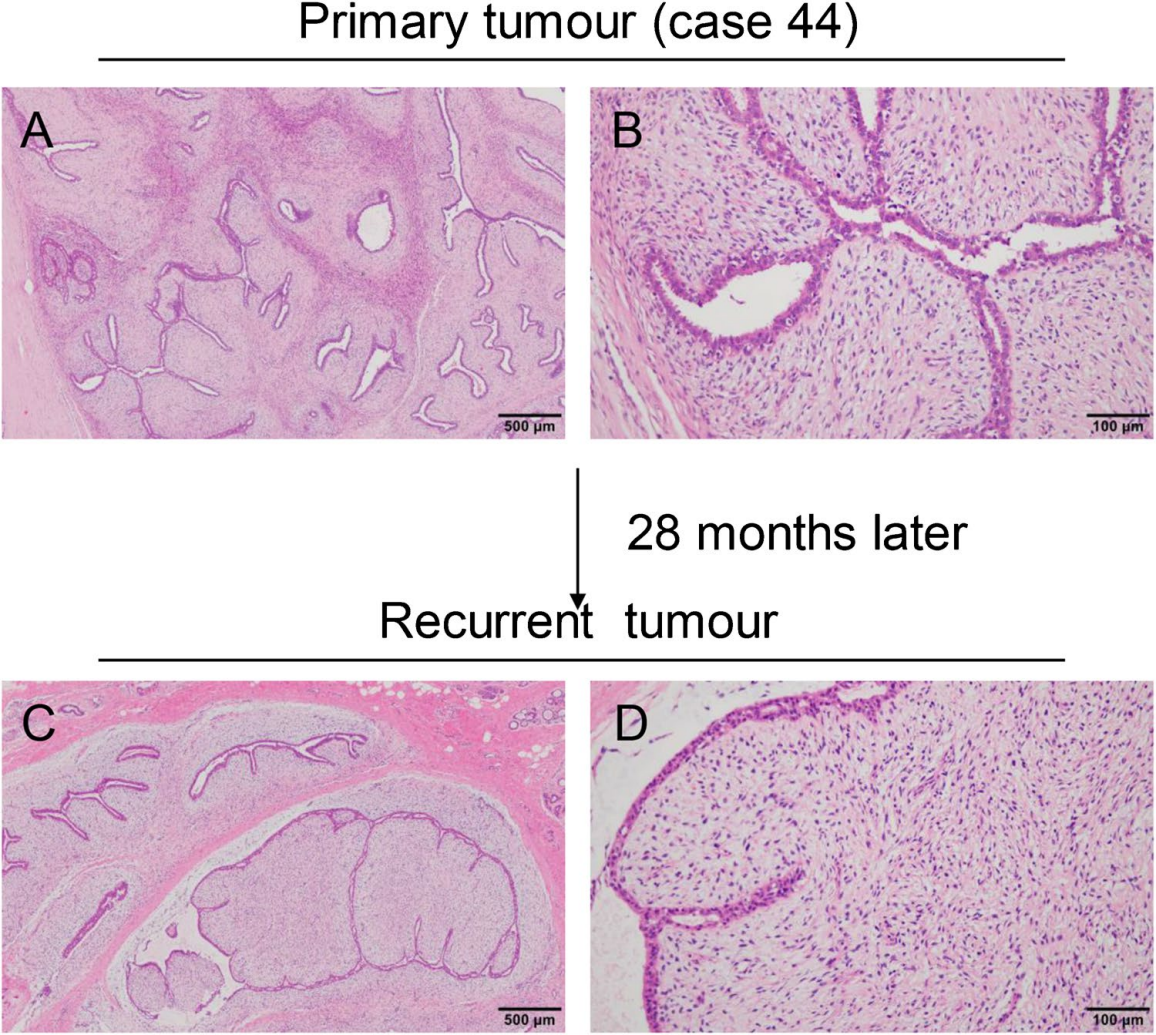
Phyllodes tumours with classic feature. Stromal hypercellularity and leaf fronds are obvious in both primary (**a** and **b**) and recurrent tumours (**c** and **d**)

Both primary and recurrent tumours of 12 cases typically exhibited features that fully met the diagnostic requirements of PTs. These tumours had exaggerated intracanalicular growth patterns, with leaf-like projections extending into variably dilated and elongated lumina, and varying degrees of cellular atypia. Permeative borders, stromal hypercellularity, atypical spindle cells, and clear mitotic figures could be observed in two borderline grade primary tumours, instead of benign grade tumours (Fig. 6). Tumour progression after recurrence occurred in seven cases.

### Associations between clinicopathological characteristics and PTs with various features

Patients diagnosed with PTs with epithelioid feature had the shortest recurrence interval time (median 10 months, range 8–14 months), while those diagnosed with PTs with PASH feature had the longest recurrence interval time (median 67.5 months, range 31–71 months). Except for PTs with epithelioid feature and myxoid feature, the original grade of tumours with other features was mainly benign. Regardless of their different features, most tumours recurred once and the tumour grade progressed in nearly half of cases after recurrence. The differences in the morphological features of PTs in each group of tumours were statistically significant (Table 2).

Compared with classic PTs, PTs with epithelioid feature and myxoid feature were more prone to showing permeative tumour border (*P* = 0.022, *P* = 0.028). PTs with epithelioid feature displayed the most remarkable cell atypia (*P* = 0.009), whereas PTs with gland-rich feature and FA-like feature showed the opposite, with bland tumour cells in the majority of cases (*P* < 0.001). The number of mitoses in PTs with PASH feature was significantly fewer than those in the classic PTs (*P* = 0.007); however, these tumours with PASH feature showed much more obvious hyalinization compared to PTs with classic features (*P* = 0.003). Enlargement of the epithelial–stromal junction was less likely to appear in PTs with epithelioid feature, myxoid feature, and PASH feature compared to PTs with the other features (*P* = 0.022, *P* = 0.003, *P* = 0.008) (Table 3; Supplementary Table 2).

**Table 3:** The comparison of various morphological parameters between PTs with classic feature and PTs with non–classic feature (*P* value)

	Classic feature and epithelia feature	Classic feature and gland-rich feature	Classic feature and FA-like feature	Classic feature and myxoid feature	Classic feature and PASH feature
Location	0.525	0.362	0.489	1.000	0.262
Frequency of recurrence	0.505	0.325	0.073	0.081	1.000
Tumour upgrade	1.000	1.000	0.716	1.000	1.000
Tumour border	0.022	1.00	0.133	0.028	1.000
Cellularity	1.000	1.000	1.000	1.000	1.000
Atypia	0.009	< 0.001	< 0.001	1.000	1.000
Mitoses	NA	0.147	0.271	NA	0.007
Enlargement of epithelial–stromal junction	0.022	0.495	0.133	0.003	0.008
Myxoid changes	1.000	0.242	1.000	0.009	0.529
Hyalinisation	1.000	1.000	0.212	1.000	0.003
Vessel proliferation	0.002	0.147	0.13	NA	< 0.001
PASH	0.505	0.325	0.726	0.245	0.088
Epithelial hyperplasia	0.002	NA	0.029	< 0.001	NA

*FA*, fibroadenoma; *PT*, phyllodes tumour; *PASH*, pseudohemangiomatoid stromal hyperplasia; *NA*, statistical analysis of this data is not applicable

### Histological features in FAs without recurrence and PTs with FA-like feature

Amongst the six variant features of primary PTs, the FA-like feature was the most common, and lead to the challenges of distinguishing it from the classic FAs. To further study this, we collected an additional 50 cases of FAs without a history of recurrence. The median age in these cases was 28 years, and the mean tumour size was 2.08 cm, 21 cases occurred on the left side, 26 cases occurred on the right side, and 3 cases on both sides. The comparison between the characteristics of these two tumours is shown in Table 4. Histologically, a predominantly intracanalicular pattern, with well-defined border and absence of leaf frond structure, was observed in both groups. PTs with FA-like feature were significantly positively associated with the presence of epithelial–stromal junction, PASH, small blood vessel proliferation, and definite mitoses, whereas typical FAs were significantly correlated with the presence of diffuse collagenisation and hyalinisation (*P* < 0.001). Moreover, more than half of the PTs with FA-like feature showed EGFR positivity.

**Table 4:** Histological features and IHC staining results of FAs without local recurrence and primary PTs with FA-like feature

	FAs without local recurrence	PTs with FA-like pattern	*P* value
	*N* = 50	*N* = 20	
Growth pattern			0.732
Intracanalicular	42	16	
Pericanalicular	8	4	
Tumour border			
Well-defined	50	20	
Permeative	0	0	
Leaf-like fronds			
Absent	50	20	
Present	0	0	
Enlargement of epithelial–stromal junction			< 0.001
Absent	50	2	
Present	0	18	
PASH			< 0.001
Absent	48	10	
Present	2 (focally)	10	
Myxoid change			0.619
Absent	47	18	
Present	3 (focally)	2 (focally)	
Collagenisation and hyalinisation			< 0.001
Absent	5	16	
Present	45	4 (focally)	
Vessel proliferation			< 0.001
Absent	47	5	
Present	3 (focally)	15	
Mitoses/mm^2^			< 0.001
Absent	50	4	
Present (≥ 1)	0	16	
UDH (ductal hyperplasia?)			0.035
Absent	32	7	
Present	18	13	
CD34 stain			0.708
Positive	44	17	
Negative	6	3	
Bcl-2 stain			0.194
Positive	1	2	
Negative	49	18	
EGFR stain			
Positive	0	16 (one diffuse, 15 focal)	< 0.001
Negative	50	4	

*FA*, fibroadenoma; *PT*, phyllodes tumour; *PASH*, pseudohemangiomatoid stromal hyperplasia; *UDH*, usual ductal hyperplasia

### Immunohistochemistry results

Immunohistochemical studies showed that PTs with epithelioid feature were negative for CD34, but diffusely positive for Bcl-2 and EGFR. Tumour cells in most other cases stained positive CD34, with the exception of PTs with myxoid feature which did not. Three cases categorised with gland-rich feature (37.5%), 16 cases categorised with FA-like feature (80%), 4 cases categorised with PASH feature (100%), and 8 cases categorised with classic feature (66.67%) displayed weak positivity EGFR in focal areas, even in tumours with benign grade (Table 5).

**Table 5:** Summary results of IHC staining of primary PTs with different features

Features		CD34		Bcl-2		EGFR	
		Positive	Negative	Positive	Negative	Positive	Negative
Epithelioid feature (%)	*N* = 3	1 (33.3%)	2 (66.7%)	3 (100%)	0	3 (100%)	0
Gland-rich feature (%)	*N* = 8	7 (87.5%)	1 (12.5%)	1 (12.5%)	7 (87.5%)	3 (37.5%)	5 (62.5%)
FA-like feature (%)	*N* = 20	17 (85%)	3 (15%)	2 (10%)	18 (90%)	16 (80%)	4 (20%)
Myxoid feature (%)	*N* = 5	1 (20%)	4 (80%)	3 (60%)	2 (40%)	0	5 (100%)
PASH feature (%)	*N* = 4	4 (100%)	0	1 (25%)	3 (75%)	4 (100%)	0
Classic feature (%)	*N* = 12	11 (91.67%)	1 (8.3%)	7 (58.3%)	5 (41.7%)	8 (66.67%)	4 (33.33%)

*FA*, fibroadenoma; *PT*, phyllodes tumour; *PASH*, pseudohemangiomatoid stromal hyperplasia

## Discussion

The diagnosis of PT and the differential diagnosis between PT and FA rely on a comparative analysis of multiple subjective histological parameters. Laura et al. performed an 11-institution contemporary review of PTs and found that there were many differences in the understanding of histopathologic features and that there were no standardized reports on the characterisation of PTs [11]. Moreover, it was found that PTs may develop in an unusual pattern-mimicking endometriosis [12], or with osteoclast-like giant cells [13]. In this study, we explored tumour heterogeneity from the early stage of tumour development. The surgical margins of the samples from the first surgical resection were all positive, and all the patients developed ipsilateral local recurrence. At present, stromal fronds and hypercellularity are considered to be essential diagnostic criteria for PTs, but some cases in this cohort of recurrent tumours did not exhibit these two features in the primary tumours. Thus, we proposed five distinct features of PTs, when compared to currently used diagnostic features of classic primary PTs: epithelioid, gland-rich, FA-like, myxoid, and PASH.

The PTs with epithelioid feature had a high tumour grade and the shortest recurrence interval time. The neoplastic stroma showed remarkable atypia and was similar to the epithelia, not to the spindle cells. However, the mixed distribution of the epithelium and the stromal and the ‘lack’ of myoepithelial cells in the H&E-stained sections may lead to the misdiagnoses of invasive carcinoma, carcinosarcoma, or lymphomas. It is, thus, necessary to use highly specific myoepithelial markers to confirm the integrity of the myoepithelium. The PTs with gland-rich feature may mimic adenosis because of the existence of TDLU and abundant glands in the tumour area. However, the expansion of the TDLU governed by the enlargement of the epithelial–stromal junction with a significant increase in the number of breast acini may assist in avoiding this confusion. The most common feature was the FA-like one. During the early stage of PTs, it was difficult to distinguish PT from FA in the first resection slide. However, after reviewing the histological features of 50 typical FA cases without recurrence, some specific characteristics were noticed. An even cell distribution and the collagenisation and hyalinisation of stromal cells were in favour of the diagnosis of FA, while the emergence of epithelial–stromal junction, small vascular proliferation, and mitoses supported the diagnosis of PT. EGFR staining in PTs with benign grade can be focally positive. The diffuse distribution of mucus in the tumour, accompanied by the formation of mucus lake, abundant small blood vessels with branches, and sparse tumour cells with no or mild atypia, is the major characteristic of PT with myxoid feature. Myxoid changes with no atypia are usually defined to be myxoid FA [14], but this study found that this type of lesion often became borderline or malignant after recurrence. The search for clear mitotic figures at high magnification is an extremely important breakthrough that can help better reveal the nature of the lesion rather than relying on cellular atypia to enable the distinction of PT from FA with myxoid change. The naive tumour cells and the branched, thin-walled vessels are also highly suggestive of PT. Moreover, tumour cells in myxoid PTs were usually negative for CD34, which may suggest that the origin of this tumour is different from that of the other five types. Another growth pattern is the PASH, in which neoplastic stromal cells proliferate with anastomosing slit-like spaces. Previous studies have suggested that PASH is a phenomenon that accompanies PTs [15, 16], and our study revealed that PASH may be one of the early structural forms of PTs. In classic PTs, hypercellularity and leaf-like fronds can be observed in both primary and recurrent tumours. Tumours of different histological grades have morphological overlaps, indicating that tumours may be in a dynamic evolutionary process.

In addition to the presence of recognisable stromal overgrowth and a leaf-like structure, some tumours possess other distinct features, such as neoplastic cells with epithelioid morphology, abundant glands, or myxoid changes, which often lead to diagnostic uncertainty and inadequate clinical treatment. Thus, in this retrospective study, we have described some additional diagnostic clue for the diagnosis of PTs. Amongst these diagnostic clues is the enlargement of the junctional area between the epithelium and stroma, which is accompanied by the presence of an abrupt boundary between intralobular stroma and interlobular stroma, and this is in line with the theory that the tumour originates from stromal cells in the lobule. Another feature is the neoplastic cells showing diffuse PASH-like proliferation accompanied by collagen production, mimicking the morphology of interlobular stroma, which may suggest that tumorigenesis is not related to only intralobular stroma. The hyperplasia of the ductal epithelium was also a useful clue because some studies have suggested that an overactive epithelium can induce the stromal cells adjacent to the glands to proliferate and develop into tumours through the activation of the WNT signalling pathway [17, 18].

In conclusion, from this group of cases with classic PT morphological changes after recurrence, it can be recognised that stromal hypercellularity and a leaf-like structure may not be present in early stage of PT but may only occur in the morphological manifestation of tumour development at a specific stage. Therefore, we recommend that these two histologic features not be over-evaluated in assessing the properties of fibroepithelial tumours and that more attention be paid to the indicators of tumour cell atypia and mitosis. However, since this study was limited by the small number of cases and a lack of molecular genetic evidence, further research is necessary.

## Supplementary Information

Below is the link to the electronic supplementary material.

Supplementary file1 (DOCX 23 KB)

Supplementary file2 (DOCX 27 KB)

Supplementary file3 (DOCX 27 KB)

## Data Availability

All data generated or analysed during this study are included in this published article.
