# Supplementary material for: Morphological features of 52 cases of breast phyllodes tumours with local recurrence
Source: Virchows Arch. 2022 Jul 29;481(4):519–31. doi: 10.1007/s00428-022-03383-8 (PMC9534817; doi:10.1007/s00428-022-03383-8)
Supplement: Supplementary file 1 — Supplementary file1 (DOCX 23 KB) [file 428_2022_3383_MOESM1_ESM.docx]

Supplementary Table 1. Detail clinicopathological information of 52 patients with PTs

| Features | Case no. | Age at 1^st^ diagnosis (year) | 1^st^ recurrence interval time (months) | Side | Size at 1^st^ diagnosis (cm) | Recurrence | Tumour grade (1^st^/2^nd^/3^rd^) |
| --- | --- | --- | --- | --- | --- | --- | --- |
| Epithelioid feature | 1 | 18 | 14 | L | 3.5 | Once | BL/M |
|  | 2 | 21 | 10 | L | 2.0 | Once | BL/M |
|  | 3 | 47 | 8 | R | 9.0 | Once | M/M |
| Gland–rich feature | 4 | 34 | 92 | R | 2.5 | Once | B/B |
|  | 5 | 22 | 20 | L | 3.5 | Once | B/BL |
|  | 6 | 28 | 80 | L | 4.5 | Once | BL/M |
|  | 7 | 40 | 15 | L | 5.0 | Twice | B/BL/M |
|  | 8 | 37 | 48 | R | 2.3 | Once | B/B |
|  | 9 | 35 | 13 | L | 3.0 | Once | BL/BL |
|  | 10 | 29 | 32 | R | 4.0 | Once | B/BL |
|  | 11 | 32 | 13 | L | 9.7 | Once | BL/M |
| FA–like feature | 12 | 34 | 95 | R | 2.0 | Once | B/BL |
|  | 13 | 33 | 3 | R | 2.5 | Once | B/B |
|  | 14 | 24 | 16 | L | 1.5 | Once | B/BL |
|  | 15 | 35 | 12 | L | 3.0 | Once | B/BL |
|  | 16 | 28 | 6 | L | 2.0 | Once | B/B |
|  | 17 | 32 | 23 | R | 3.5 | Once | B/BL |
|  | 18 | 36 | 24 | L&R | 1.5 (L), 2.5 (R) | Once | B/B |
|  | 19 | 29 | 12 | R | 5.0 | Once | BL/BL |
|  | 20 | 20 | 9 | R | 4.0 | Once | BL/BL |
|  | 21 | 46 | 25 | R | 2.0 | Twice | B/BL/M |
|  | 22 | 33 | 20 | L | 3.0 | Once | B/B |
|  | 23 | 26 | 21 | R | 3.5 | Once | B/B |
|  | 24 | 19 | 13 | R | 5.5 | Once | B/BL |
|  | 25 | 19 | 64 | R | 2.0 | Once | B/B |
|  | 26 | 26 | 89 | R | 4.5 | Once | B/B |
|  | 27 | 26 | 89 | L&R | 2.0 (L), 2.0 (R) | Once | B/B |
|  | 28 | 36 | 7 | R | 5.5 | Once | B/BL |
|  | 29 | 40 | 5 | R | 4.5 | Twice | BL/M/M |
|  | 30 | 27 | 25 | R | 1.7 | Once | B/M |
|  | 31 | 39 | 23 | R | 4.0 | Once | B/B |
| Myxoid feature | 32 | 50 | 82 | L | 2.2 | Once | B/BL |
|  | 33 | 35 | 26 | R | 6.0 | Once | BL/M |
|  | 34 | 35 | 11 | L | 1.5 | Thrice | B/B/BL/BL |
|  | 35 | 36 | 38 | R | 1.0 | Once | BL/BL |
|  | 36 | 37 | 21 | R | 5.0 | Once | BL/BL |
| PASH feature | 37 | 33 | 65 | L | 2.6 | Once | B/BL |
|  | 38 | 28 | 31 | R | 1.3 | Twice | B/BL/BL |
|  | 39 | 39 | 70 | L | 3.0 | Once | B/B |
|  | 40 | 26 | 71 | L | 2.5 | Once | B/B |
| Classic feature | 41 | 25 | 4 | R | 2.0 | Once | B/B |
|  | 42 | 51 | 5 | L | 1.2 | Twice | BL/BL/M |
|  | 43 | 38 | 40 | R | 2.5 | Twice | B/B/BL |
|  | 44 | 42 | 28 | L | 3.0 | Once | B/BL |
|  | 45 | 22 | 105 | R | 4.0 | Once | B/B |
|  | 46 | 31 | 41 | L | 2.0 | Twice | B/B/BL |
|  | 47 | 22 | 25 | R | 3.0 | Once | B/BL |
|  | 48 | 25 | 114 | R | 1.2 | Once | B/B |
|  | 49 | 22 | 45 | L | 3.5 | Twice | B/B/BL |
|  | 50 | 30 | 26 | R | 2.5 | Once | B/B |
|  | 51 | 16 | 12 | R | 4.0 | Twice | B/BL/BL |
|  | 52 | 22 | 105 | R | 2.3 | Once | BL/BL |

FA, fibroadenoma; PT, phyllode tumour; PASH, pseudo hemangiomatoid stromal hyperplasia; L, left; R, right; B, benign; BL, borderline; M, malignant.
