# Supplementary material for: Morphological features of 52 cases of breast phyllodes tumours with local recurrence
Source: Virchows Arch. 2022 Jul 29;481(4):519–31. doi: 10.1007/s00428-022-03383-8 (PMC9534817; doi:10.1007/s00428-022-03383-8)
Supplement: Supplementary file 2 — Supplementary file2 (DOCX 27 KB) [file 428_2022_3383_MOESM2_ESM.docx]

Supplementary Table 2. Pathological review of primary tumours of 52 patients with PTs

|  | Case no. | Tumour border | Cellularity | Atypia | Overgrowth | Mitotic count (per mm^2^) | Enlargement of epithelial-stromal junction | Leaf–like fronds | Myxoid changes | Hyalinisation | Vessel proliferation | PASH | Epithelial hyperplasia |
| --- | --- | --- | --- | --- | --- | --- | --- | --- | --- | --- | --- | --- | --- |
| Epithelioid feature | 1 | Permeative | Mild | Moderate | Absent | 3.1 | Absent | Absent | Absent | Absent | Absent | Absent | Absent |
|  | 2 | Permeative | Mild | Moderate | Absent | 4.6 | Absent | Absent | Absent | Absent | Absent | Absent | Absent |
|  | 3 | Permeative | Mild | Marked | Absent | 6.9 | Absent | Absent | Absent | Absent | Absent | Absent | Absent |
| Gland–rich feature | 4 | Well defined | Mild | None | Absent | 0.4 | Present focally | Absent | Absent | Absent | Present focally | Absent | Present |
|  | 5 | Well defined | Mild | None | Absent | 0.4 | Present focally | Absent | Absent | Absent | Present focally | Absent | Present |
|  | 6 | Well defined | Mild | None | Absent | 1.9 | Present focally | Absent | Absent | Absent | Present | Absent | Present |
|  | 7 | Well defined | Mild | Mild | Absent | 0.8 | Present focally | Absent | Absent | Absent | Present focally | Absent | Present |
|  | 8 | Well defined | Mild | None | Absent | 0 | Present focally | Absent | Absent | Absent | Absent | Absent | Present |
|  | 9 | Focally permeative | Moderate | Moderate | Absent | 2.7 | Present focally | Absent | Absent | Absent | Present | Absent | Present |
|  | 10 | Well defined | Mild | None | Absent | 0 | Present focally | Absent | Absent | Absent | Absent | Present focally | Present |
|  | 11 | Well defined | Moderate | Moderate | Absent | 2.3 | Present | Absent | Absent | Absent | Present | Absent | Present |
| FA–like feature | 12 | Well defined | Mild | None | Absent | 0.4 | Present focally | Absent | Absent | Absent | Present | Present focally | Present |
|  | 13 | Well defined | Mild | None | Absent | 0.4 | Present focally | Absent | Absent | Absent | Present | Present focally | Present |
|  | 14 | Well defined | Mild | Mild | Absent | 0.8 | Present focally | Absent | Absent | Absent | Present | Present focally | Present |
|  | 15 | Well defined | Mild | Mild | Absent | 0 | Present focally | Absent | Present focally | Present focally | Absent | Present focally | Absent |
|  | 16 | Well defined | Mild | None | Absent | 0.8 | Present focally | Absent | Absent | Absent | Present | Absent | Present |
|  | 17 | Well defined | Mild | None | Absent | 0 | Present focally | Absent | Present focally | Present focally | Absent | Present focally | Absent |
|  | 18 | Well defined | Mild | None | Absent | 0 | Present focally | Absent | Absent | Present focally | Present | Absent | Absent |
|  | 19 | Well defined | Moderate | Mild | Absent | 2.3 | Present | Absent | Absent | Absent | Present | Absent | Present |
|  | 20 | Well defined | Moderate | Mild | Absent | 1.2 | Present | Absent | Absent | Absent | Present | Present focally | Present |
|  | 21 | Well defined | Mild | None | Absent | 0.8 | Present | Absent | Absent | Absent | Present | Absent | Absent |
|  | 22 | Well defined | Mild | None | Absent | 0 | Present focally | Absent | Present focally | Present focally | Absent | Absent | Absent |
|  | 23 | Well defined | Mild | None | Absent | 0.4 | Present focally | Absent | Absent | Present focally | Absent | Present focally | Absent |
|  | 24 | Well defined | Mild | Mild | Absent | 0.4 | Present | Absent | Present focally | Present focally | Absent | Present focally | Present |
|  | 25 | Well defined | Mild | None | Absent | 0.4 | Present focally | Absent | Present focally | Absent | Present | Absent | Present |
|  | 26 | Well defined | Mild | None | Absent | 0.8 | Present focally | Absent | Absent | Absent | Present | Absent | Present |
|  | 27 | Well defined | Mild | Mild | Absent | 1.2 | Present focally | Absent | Absent | Absent | Present | Absent | Present |
|  | 28 | Well defined | Mild | None | Absent | 0.4 | Present | Absent | Absent | Absent | Present | Absent | Absent |
|  | 29 | Well defined | Moderate | Mild | Absent | 3.1 | Present | Absent | Absent | Absent | Present | Absent | Present |
|  | 30 | Well defined | Mild | None | Absent | 1.2 | Present | Absent | Absent | Absent | Present | Present focally | Present |
|  | 31 | Well defined | Mild | None | Absent | 0.4 | Present focally | Absent | Absent | Absent | Present | Present focally | Present |
| Myxoid feature | 32 | Well defined | Mild | Mild | Absent | 0.4 | Absent | Absent | Present | Absent | Present focally | Absent | Absent |
|  | 33 | Focally permeative | Mild | Mild | Absent | 1.2 | Absent | Absent | Present | Absent | Present | Absent | Absent |
|  | 34 | Permeative | Mild | Mild | Absent | 0.8 | Absent | Absent | Present | Absent | Present | Absent | Absent |
|  | 35 | Permeative | Mild | Mild | Absent | 0.8 | Absent | Absent | Present | Absent | Present | Absent | Absent |
|  | 36 | Permeative | Mild | Mild | Absent | 0.4 | Absent | Absent | Present | Absent | Present | Absent | Absent |
| PASH feature | 37 | Well defined | Mild | Mild | Absent | 0.4 | Absent | Absent | Absent | Present | Absent | Present | Present |
|  | 38 | Permeative focally | Mild | Mild | Absent | 0.4 | Present focally | Absent | Absent | Present | Absent | Present | Present |
|  | 39 | Well defined | Mild | Mild | Absent | 0.4 | Absent | Absent | Absent | Present | Absent | Present | Present |
|  | 40 | Well defined | Mild | Mild | Absent | 0 | Absent | Absent | Absent | Present | Absent | Present | Present |
| Classic feature | 41 | Well defined | Mild | Mild | Absent | 0.8 | Absent | Present | Present focally | Absent | Present | Present focally | Present |
|  | 42 | Permeative | Moderate | Moderate | Absent | 2.3 | Absent | Present | Absent | Absent | Present | Present focally | Present |
|  | 43 | Well defined | Mild | Mild | Absent | 0.8 | Present | Present | Absent | Absent | Present | Absent | Present |
|  | 44 | Well defined | Mild | Mild | Absent | 0.8 | Present | Present | Absent | Present focally | Present | Present focally | Present |
|  | 45 | Well defined | Mild | Mild | Absent | 0.4 | Present | Present | Absent | Absent | Present | Absent | Present |
|  | 46 | Well defined | Mild | Mild | Absent | 0.8 | Present | Present | Absent | Absent | Present | Absent | Present |
|  | 47 | Well defined | Mild | Mild | Absent | 0.8 | Present | Present | Absent | Absent | Present | Absent | Present |
|  | 48 | Well defined | Mild | Mild | Absent | 1.2 | Present | Present | Absent | Absent | Present | Absent | Present |
|  | 49 | Well defined | Mild | Mild | Absent | 0.8 | Present | Present | Present focally | Absent | Present | Present focally | Present |
|  | 50 | Well defined | Mild | Mild | Absent | 1.2 | Present | Present | Present focally | Absent | Present | Present focally | Present |
|  | 51 | Well defined | Mild | Mild | Absent | 1.2 | Present | Present | Absent | Absent | Present focally | Absent | Present |
|  | 52 | Permeative | Moderate | Mild | Absent | 2.7 | Present | Present | Absent | Absent | Present | Absent | Present |

PT, phyllode tumour; PASH, pseudo hemangiomatoid stromal hyperplasia; B, benign; BL, borderline; M, malignant.
