# Supplementary material for: Morphological features of 52 cases of breast phyllodes tumours with local recurrence
Source: Virchows Arch. 2022 Jul 29;481(4):519–31. doi: 10.1007/s00428-022-03383-8 (PMC9534817; doi:10.1007/s00428-022-03383-8)
Supplement: Supplementary file 3 — Supplementary file3 (DOCX 27 KB) [file 428_2022_3383_MOESM3_ESM.docx]

Supplementary Table 3 CD34, Bcl-2, and EGFR expression in PTs with recurrence

|  | Case no. | CD34 | | Bcl-2 | | EGFR | | Tumour grade (1^st^/2^nd^/3^rd^) |
| --- | --- | --- | --- | --- | --- | --- | --- | --- |
|  |  | Primary | Recurrence | Primary | Recurrence | Primary | Recurrence |  |
| Epithelioid feature | 1 | N | N | D | D | D | D | BL/M |
|  | 2 | N | N | D | D | D | D | BL/M |
|  | 3 | F | N | F | D | D | D | M/M |
| Gland–rich feature | 4 | D | D | N | N | N | N | B/B |
|  | 5 | D | D | N | N | N | N | B/BL |
|  | 6 | D | D | N | N | F | D | BL/M |
|  | 7 | D | D | N | F | N | D | B/BL/M |
|  | 8 | D | D | N | N | N | N | B/B |
|  | 9 | D | D | N | N | F | F | BL/BL |
|  | 10 | D | D | N | F | N | N | B/BL |
|  | 11 | N | N | D | D | D | D | BL/M |
| FA–like feature | 12 | N | D | N | F | N | F | B/BL |
|  | 13 | D | D | N | N | F | F | B/B |
|  | 14 | D | D | N | F | F | F | B/BL |
|  | 15 | D | N | D | D | F | F | B/BL |
|  | 16 | D | D | N | N | F | F | B/B |
|  | 17 | D | N | N | D | F | D | B/BL |
|  | 18 | D | D | N | N | F | F | B/B |
|  | 19 | D | D | N | F | F | D | BL/BL |
|  | 20 | D | D | D | D | F | D | BL/BL |
|  | 21 | D | D | N | F | F | D | B/BL/M |
|  | 22 | D | D | N | N | F | F | B/B |
|  | 23 | D | D | N | N | N | F | B/B |
|  | 24 | D | D | N | F | F | F | B/BL |
|  | 25 | D | D | N | N | N | F | B/B |
|  | 26 | D | D | N | F | F | F | B/B |
|  | 27 | D | D | N | N | F | F | B/B |
|  | 28 | N | D | N | D | N | F | B/BL |
|  | 29 | N | N | N | D | D | D | BL/M/M |
|  | 30 | D | N | N | D | F | D | B/M |
|  | 31 | D | D | N | N | F | F | B/B |
| Myxoid feature | 32 | N | N | N | F | N | N | B/BL |
|  | 33 | F | N | F | N | N | N | BL/M |
|  | 34 | N | N | N | N | N | N | B/B/BL/BL |
|  | 35 | N | N | F | F | N | N | BL/BL |
|  | 36 | N | N | F | F | N | N | BL/BL |
| PASH feature | 37 | D | N | N | D | F | F | B/BL |
|  | 38 | D | D | D | D | F | F | B/BL/BL |
|  | 39 | D | D | N | N | F | F | B/B |
|  | 40 | D | D | N | N | F | F | B/B |
| Classic feature | 41 | D | D | N | N | N | F | B/B |
|  | 42 | N | N | N | D | D | D | BL/BL/M |
|  | 43 | D | N | N | D | F | F | B/B/BL |
|  | 44 | D | D | N | D | F | F | B/BL |
|  | 45 | D | D | N | N | N | F | B/B |
|  | 46 | D | D | N | D | N | D | B/B/BL |
|  | 47 | D | N | D | D | F | F | B/BL |
|  | 48 | D | D | N | D | F | F | B/B |
|  | 49 | D | D | N | N | F | F | B/B/BL |
|  | 50 | D | D | N | N | N | F | B/B |
|  | 51 | D | D | N | D | F | F | B/BL/BL |
|  | 52 | D | D | N | N | F | F | BL/BL |

FA, fibroadenoma; PT, phyllode tumour; PASH, pseudohemangiomatoid stromal hyperplasia; B, benign; BL, borderline; M, malignant; IHC, immunohistochemistry; Bcl2, B cell leukemia/lymphoma 2; EGFR, epidermal growth factor receptor; F, focally positive; D, diffusely positive; N, negative.
